# Daily rhythms of cloacal temperature in broiler chickens of different age groups administered with zinc gluconate and probiotic during the hot‐dry season

**DOI:** 10.14814/phy2.13314

**Published:** 2017-06-22

**Authors:** Tagang Aluwong, Victory O. Sumanu, Joseph O. Ayo, Benjamin O. Ocheja, Friday O. Zakari, Ndazo S. Minka

**Affiliations:** ^1^ Department of Veterinary PhysiologyFaculty of Veterinary MedicineAhmadu Bello UniversityZariaNigeria; ^2^ Division of Agricultural CollegesCollege of Agriculture and Animal ScienceAhmadu Bello UniversityMando‐KadunaNigeria

**Keywords:** Broiler chickens, heat stress, hot‐dry season, probiotic, zinc gluconate

## Abstract

The aim of the experiment was to evaluate effects of zinc gluconate (ZnGlu) and probiotic administration on the daily rhythm of cloacal temperature (*t*_cloacal_) in broiler chickens of different age groups during the hot‐dry season. One‐day‐old broiler chicks (*n* = 60) were divided into groups I–IV of 15 chicks per group, and treated for 35 days: Group I (control) was given deionized water; Group II, ZnGlu (50 mg/kg); Group III, probiotic (4.125 × 10^6 ^cfu/100 mL), and Group IV, ZnGlu (50 mg/kg) + probiotic (4.125 × 10^6 ^cfu/100 mL). Air dry‐bulb temperature (*t*_db_), relative humidity (RH), and temperature‐humidity index (THI) inside the pen, and *t*_cloacal_ of each broiler chick were obtained bihourly over a 24‐h period; on days 21, 28, and 35 of the study. Values of t_db_ (32.10 ± 0.49°C), RH (49.94 ± 1.91%), and THI (38.85 ± 0.42) obtained were outside the thermoneutral zone for broiler chickens, and suggested that the birds were subjected to heat stress. Application of the periodic model showed disruption of daily rhythm of *t*_cloacal_ in broilers on day 21, which was synchronized by probiotic administration. The administration of probiotics or ZnGlu + probiotics to a greater extent decreased the mesor and amplitude, delayed the acrophases of *t*_cloacal_ in broilers, especially at day 35, as compared to the controls. Overall, the *t*_cloacal_ values in broiler chickens administered with probiotic alone (41.25 ± 0.05°C) and ZnGlu + probiotic (41.52 ± 0.05°C) were lower (*P* < 0.001) than that of the controls (41.94 ± 0.06°C). In conclusion, probiotic alone synchronized *t*_cloacal_ of the birds at day 21, and, in addition, decreased *t*_cloacal_ response most, followed by its coadministration with ZnGlu, the antioxidants may be beneficial in modulating daily rhythmicity of t_cloacal_ and alleviating adverse effects of heat stress on broiler chickens during the hot‐dry season.

## Introduction

The thermal environmental conditions during the hot‐dry season in the Northern Guinea Savannah zone of Nigeria, which prevails from March to May (Dzenda et al. 2011) induce heat stress in pullets (Sinkalu and Ayo 2008), and directly exert adverse effects on the health and welfare of birds (Minka and Ayo 2013; Sinkalu et al. 2015a). Exposure to heat stress affects the circadian rhythms of many physiological variables in livestock, which may disorganize the circadian system, and consequently the productivity, welfare, and health status of animals (Ayo et al. 1998; Piccione et al. 2013; Minka and Ayo 2016b). The thermoneutral zone for poultry is 18–24°C in the tropics (Dei and Bumbie 2011), but the upper limit of this range is often exceeded in the tropics. In the tropics, the thermoneutral zone for broilers that are 5‐week old is 18–24°C; whereas for broilers that are 1‐, 2‐, 3‐, and 4‐week old, the thermoneutral zones are 29–33°C, 30–29.5°C, 26–28.5°C, and 23–27°C, respectively (Meltzer 1983; Scheele et al. 2014). High air dry‐bulb temperature (*t*_db_) and high relative humidity (RH), characteristic of the hot‐humid season result in heat stress (Dei and Bumbie 2011). High *t*_db_ decreases feed intake, live weight gain, and feed efficiency in broiler chickens (Niu et al. 2009; Azad et al. 2010a), and egg production in laying hens (Franco‐Jimenez and Beck 2007; Ajakaiye et al. 2010). It increases cloacal temperature (*t*_cloacal_) responses (Chowdhury et al. 2012a,b; Egbuniwe et al. 2015) and may cause heat stress in broiler chickens (Soleimani et al. 2010; Singh et al. 2015). After 21 days of age, heat stress causes mortality rate of up to 92.4% (Vale et al. 2010); and high susceptibility, which persists until the broiler chickens attain market age, at days 35–42 (Chepete et al. 2005). Thus, thermal sensitivity of broiler chickens to high t_db_ increases with a rise in body weight (Lin et al. 2004a).

Combating heat stress remains a challenge for the broiler industry in the tropics, which is even aggravated by the changing climatic conditions. The development of novel dietary measures may be beneficial in ameliorating heat stress and enhancing optimum performance in broiler chickens. Harmful effects of different stressors acting on broiler chickens during the hot‐dry season may be ameliorated by supplementing the diet of the birds with antistress agents (Erwan et al. 2012), possessing also some antioxidant activity, such as zinc gluconate (ZnGlu) and probiotic (Hasan et al. 2015), which are shown to suppress oxidative stress, and improve the health and growth performance of broiler chickens (Aluwong et al. 2013; Hasan et al. 2015). The effects of ZnGlu alone and its coadministration with probiotics on daily rhythm of *t*_cloacal_ in broiler chickens, reared during the hot‐dry season, have not been investigated. The *t*_cloacal_ is one of the indices of heat stress, reflecting the core body temperature. It indicates the balance between heat loss and heat gain in broiler chickens (Edgar et al. 2013). Thus, changes in *t*_cloacal_ during heat stress are used to evaluate the degree of adaptation of broiler chickens to hot‐dry conditions (Chen et al. 2013), and level of reactive oxygen species (ROS) in broilers (Azad et al. 2010b), generated in excess during heat stress (Lin et al. 2000).

The aim of this study was to investigate effects of ZnGlu and/or probiotic administration on daily rhythms of t_cloacal_ in broiler chickens of different age groups during the hot‐dry season.

## Materials and Methods

### Experimental site and thermal environmental conditions

The experiment was conducted at the Department of Physiology, Faculty of Veterinary Medicine, Ahmadu Bello University, Zaria (11^°^10^/^N, 07^°^ 38^/^E, altitude 686 m), located in the Northern Guinea Savannah zone of Nigeria. The broiler chickens after brooding period were kept under natural conditions, without artificial control of the microenvironment. They were, thus, subjected to the naturally‐prevailing thermal environmental conditions of high t_db_ and high RH, characteristic of the peak of the hot‐dry season in the zone; in April‐May, 2015 (Ayo et al. 2011; Dzenda et al. 2011).

### Experimental birds, management, and administration of zinc gluconate and probiotic

A total of 60, apparently, healthy broiler chickens (Arbor Acres), comprising both sexes, were used for the experiment. They were kept under an intensive management system in a standard poultry pen, littered with wood shavings. The broiler chickens were given access to water and feeds ad libitum. They were fed with commercial broiler starter (day 0–28) and broiler finisher (day 29–35), produced by Grand Cereals Limited, Jos, Nigeria. The poultry house was made of concrete floor and cement block with aluminum roofing and cardboard ceiling. The dimensions of the pen were 8.4 m × 5.6 m × 1.91 m, and the broiler chickens were stocked at 15 birds/m^2^ (Muniz et al. 2006) in order to obtain higher production volumes. The broiler chickens were randomly divided into four groups (I–IV) of 15 birds each: Control (I), ZnGlu (II), probiotic (III), and combination of probiotic + ZnGlu (IV). Both probiotic and ZnGlu were administered daily to the birds individually using a 1 mL‐tuberculin syringe for 35 days by the oral route, starting at 1‐day old. Each broiler chick was tagged, using a masking tape, on the leg for identification and proper recordings. The study was approved by the Ahmadu Bello University Committee on Animal Welfare and Use, and the management system adhered to the new European Union (EU) council directive 2007/43/EC of laying down minimum rules for the protection of chickens, kept for meat production (European Commission, 2007).

Zinc gluconate 70 mg (PHARMEDIC JSC: Ho Chi Minh City, Vietnam) was dissolved in 50 mL of deionized water and administered at a dosage of 50 mg/kg (NRC, 1994), whereas 1.5 mL/L of the probiotic (*Saccharomyces cerevisiae*) (Montajat Pharmaceuticals, Biosciences Division, Dammam 31491, Saudi Arabia) was administered daily at the concentration of 4.125 × 10^6 ^cfu/100 mL, using the competitive exclusion method for 1 week, and according to the manufacturers.

### Experimental measurements

#### Thermal environmental parameters

The *t*_dbs_ inside the pen were measured by a dry‐ and wet‐bulb thermometer (Brannan^(R)^, Cumbria, England), and the RH was calculated using Osmon's hygrometric table (Narinda Scientific Industries, Haryana, India). The *t*_db_ and RH were recorded every 2 h daily for 3 days; 1 week apart, on days 21, 28, and 35 of the experiment. The thermal environmental parameters were recorded inside the poultry house on each day of the experiment. The temperature‐humidity index (THI) for the broiler chickens was determined using the following formula (Tao and Xin 2003):

$$\text{THI}=0.85\left(t_{\mathrm{db}}\right)+0.15\left(t_{\mathrm{wb}}\right)$$

where THI = temperature‐humidity index for broilers, *t*_db_ = dry‐bulb temperature (°C) and *t*_wb_ = wet‐bulb temperature (°C).

#### Measurements of cloacal temperature

The t_cloacal_ values were recorded as an indicator of the core body temperature (Sinkalu et al. 2015b), with the aid of a digital clinical thermometer (KRAUSE digital thermometer^(R)^, DK‐5550, Langeskov, Denmark). The *t*_cloacal_ was measured, concurrently with the thermal environmental parameters for 3 days only, 1 week apart, in order to reduce the adverse effect of stress due to handling on the birds, known to increase the body temperature (Edgar et al. 2013). On each day of the recording, measurements of *t*_cloacal_ were taken 12 times using standard procedures (Minka and Ayo 2013) over a 22‐h period. After gentle catching and restraining the birds, the *t*_cloacal_ of each bird was taken by inserting the thermometer about 3‐cm deep into the cloaca for 2 min and tilting it to ensure direct contact with the wall of the cloaca. The values of thermal environment and *t*_cloacal_ were recorded concurrently and bihourly from 07:00 to 05:00 h (GMT + 1) on days 21, 28, and 35 of the study.

### Statistical analysis

Data obtained were expressed as mean ± standard error of the mean (Mean ± SEM). Cosinor analysis was used to determine the *t*_cloacal_ daily rhythms of individual birds. The mean mesor (rhythm‐adjusted mean), amplitude (half the range of excursion or a measure of the extent of predictable change within a cycle) and acrophase (time of peak) values of the variables of daily rhythm were calculated for each bird and for each time series of the study period. Values were subjected to repeated‐measures one‐way analysis of variance (ANOVA model‐3) and by the cosinor procedure (Refinetti et al. 2007; Piccione et al. 2013), followed by Tukey's multiple comparison post hoc test, using GraphPad Prism 4.0 for windows (GraphPad Software, San Diego, CA) to compare the differences between the means, obtained from the control and treated broilers. Values of *P* ˂ 0.05 were considered significant.

## Results

### Variations in thermal environmental parameters on selected days of the study period

**Figure 1:**
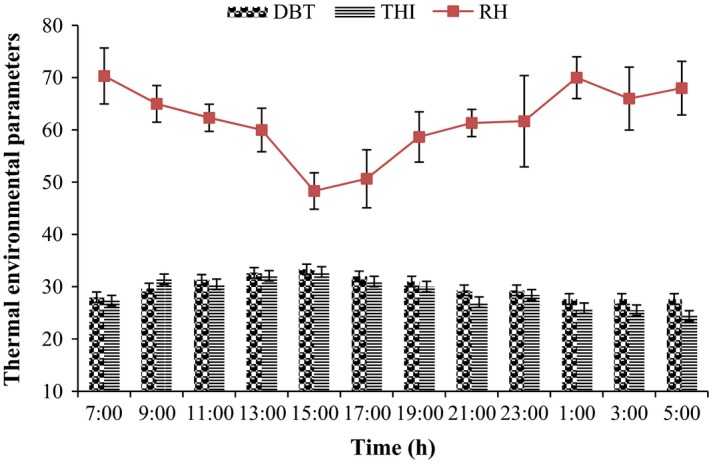
Variations in thermal environmental parameters on selected days of the study period. (*n* = 3). Significant value is at *P* < 0.05. Each data point represents the mean ± SEM of 60 birds at each period of measurements. DBT, dry‐bulb temperature; RH, relative humidity; THI, temperature‐humidity index.

The *t*_db_ on day 28 varied between (29.00–36.00°C) of the study period, and was not different, compared to the value obtained on day 21 (27.00–34.00°C) or 35 (27.00–35.00°C). There was no significant difference in RH and THI values between days 21, 28, and 35 of the study (Fig. 1). From 13:00 h to 15:00 h, *t*_db_ and THI varied from 31 to 34°C and 30.30–34.40, respectively (Table 1).

**Table 1:** Variations in thermal environmental parameters on selected days of the study period

Hours	DBT (°C)	RH (%)	THI
7:00	27–28	60–78	26.60–28.40
9:00	28–31	61–72	29.10–35.10
11:00	30–33	58–67	29.30–32
13:00	31–34	54–68	30.30–34
15:00	32–34	42–54	30.40–34.40
17:00	30–34	42–61	29.10–33.50
19:00	30–32	52–68	29.0–30.80
21:00	28–31	57–66	23.90–30
23:00	28–31	51–79	27.60–29.80
1:00	27–28	66–78	23.80–27.30
3:00	27–29	59–78	23.90–26.60
5:00	27–29	61–78	22.80–26.60
Overall mean ± SEM	29.97 ± 0.59	61.86 ± 1.99	28.89 ± 0.81

Values in parenthesis are minimum and maximum.

DBT, dry‐bulb temperature; RH, relative humidity; THI, temperature‐humidity index.

### Variations in cloacal temperature of 21‐day‐old broiler chickens during the hot‐dry season

**Figure 2:**
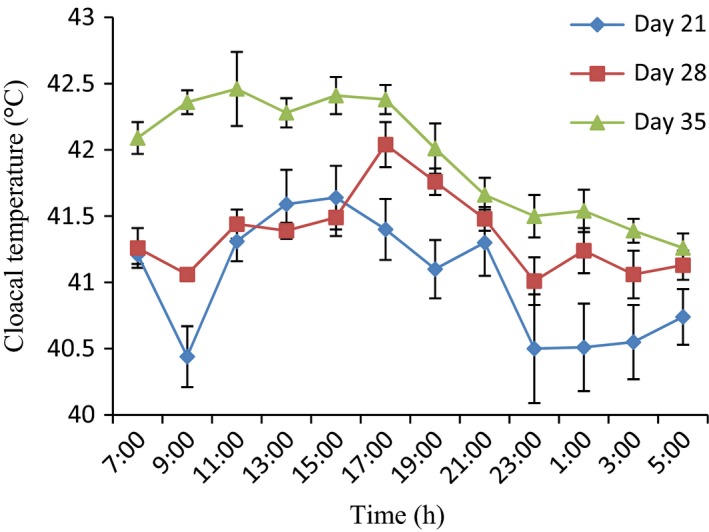
Variations in cloacal temperature of control broiler chickens at days 21, 28, and 35. (*n* = 12). Each data point represents the mean ± SEM of 60 birds at each period of measurements. The CT was higher during the day time at day 21 and decreased during the night time. For each period, measurements were taken at 2‐h intervals for a period of 24 h

The application of the periodic model showed that the t_cloacal_ of the broilers on day 21 did not exhibit a clear daily rhythm, except for broilers administered with probiotic (Fig. 4). The characteristics of cloacal temperature daily rhythms in broiler chickens of different age groups administered with ZnGlu, probiotic and ZnGlu + probiotic during the hot‐dry seasons are shown in Table 2. Although the amplitude of the *t*_cloacal_ was greater (*P* < 0.05) in control (2.20 ± 0.40°C) as compared to probiotic and ZnGlu + probiotic (0.7–1.6°C) groups, the mesor was lower (*P* < 0.05) in the control group, whereas the acrophases (13:00–15:00 h) were similar in all the groups (Fig. 2).

**Table 2:** Characteristics of cloacal temperature daily rhythms in broiler chickens of different age groups administered with ZnGlu, probiotics, and ZnGlu + probiotics during the hot‐dry seasons. For each group and on each day measurements were made at 2‐h intervals for a period of 24 h

Groups	Mesor (°C)	Amplitude (°C)	Acrophase (h) (CT°C)
	Day 21		
Control	39.90 ± 0.5^a^	2.20 ± 0.40^a^	15:00 ± 1.40^a^ (42.10)
ZnGlu	41.15 ± 0.2^b^	1.15 ± 0.40^b^	13:00 ± 1.70^a^ (42.30)
Probiotics	41.20 ± 0.2^b^	0.70 ± 0.03^c^	14:00 ± 0.90^b^ (41.90)
ZnGlu + probiotics	40.50 ± 0.4^a^	1.60 ± 0.60^d^	15:00 ± 1.10^a^ (42.10)
	Day 28		
Control	41.35 ± 0.5^a^	1.25 ± 0.10^a^	17:00 ± 1.20^a^ (42.40)
ZnGlu	40.75 ± 0.7^b^	1.15 ± 0.10^a^	17:00 ± 0.90^a^ (42.10)
Probiotics	40.85 ± 0.3^b^	0.85 ± 0.04^b^	15:00 ± 0.90^b^ (41.70)
ZnGlu + probiotics	41.15 ± 0.4^b^	0.75 ± 0.02^b^	15:00 ± 1.40^b^ (41.90)
	Day 35		
Control	42.20 ± 0.5^a^	1.55 ± 0.20^a^	11:00 ± 1.20^a^ (43.60)
ZnGlu	41.70 ± 0.5^b^	1.30 ± 0.20^b^	11:00 ± 1.40^a^ (43.00)
Probiotics	41.35 ± 0.4^c^	1.05 ± 0.10^c^	13:00 ± 1.40^b^ (42.10)
ZnGlu + probiotics	41.65 ± 0.7^b^	0.95 ± 0.05^c^	19:00 ± 1.50^c^ (42.60)

Mean values for each day with different superscript letters along the same row are significantly different at *P *<* *0.05; CT, Cloacal temperature.

### Variations in cloacal temperature of 28‐day‐old broiler chickens during the hot‐dry season

**Figure 3:**
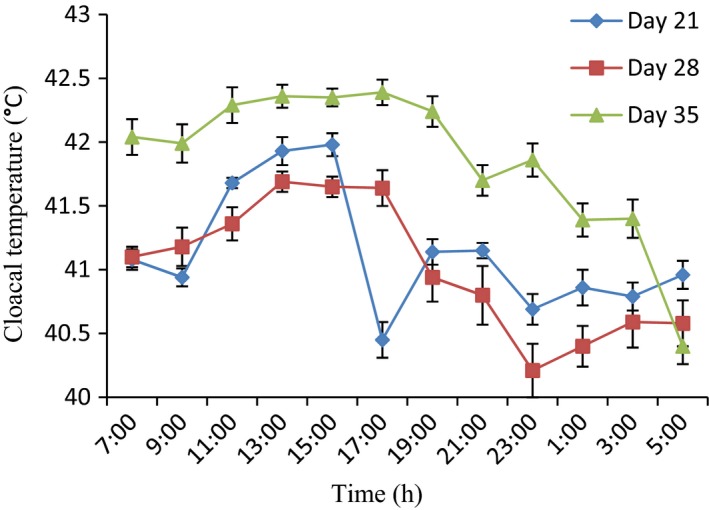
Variations in cloacal temperature of broiler chickens administered with zinc gluconate (ZnGlu) at days 21, 28, and 35. (*n* = 12). Each data point represents the mean ± SEM of 60 birds at each period of measurements. The CT was higher during the day time at day 35 and decreased during the night time.

**Figure 4:**
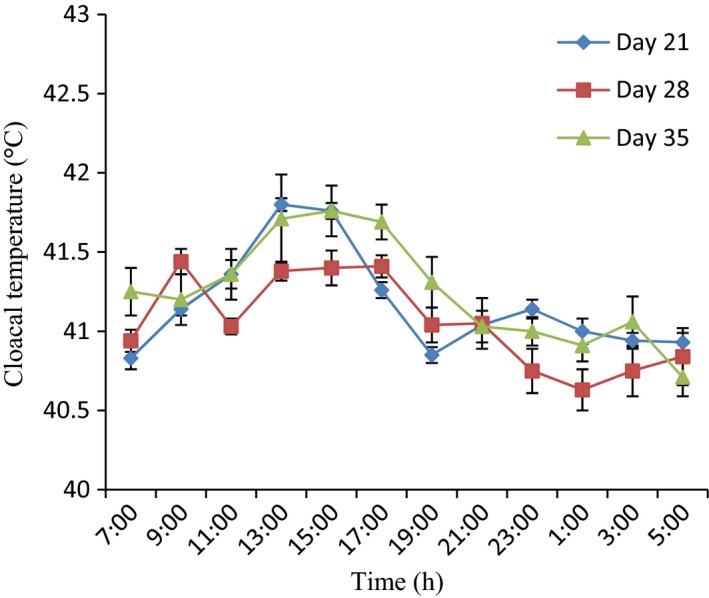
Variations in cloacal temperature of broiler chickens administered with probiotic at days 21, 28, and 35. (*n* = 12). Each data point represents the mean ± SEM of 60 birds at each period of measurements. Probiotic decreased CT responses, especially at day 28.

**Figure 5:**
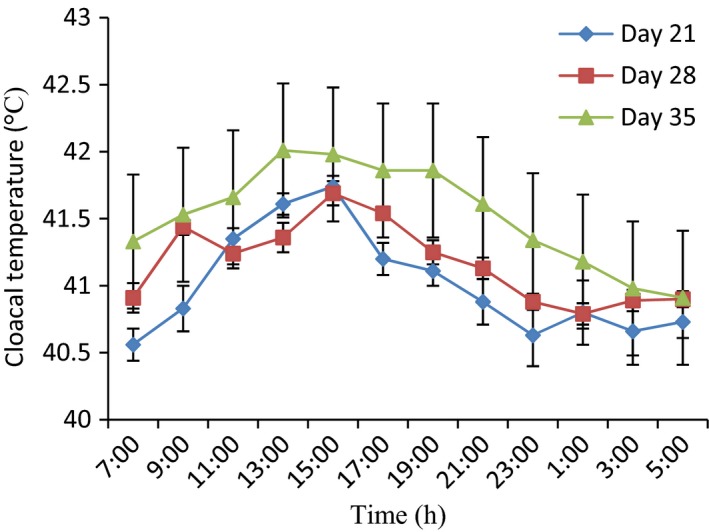
Variation in cloacal temperature of broiler chickens administered with zinc gluconate (ZnGlu) + probiotic at days 21, 28, and 35. (*n* = 12). Each data point represents the mean ± SEM of 60 birds at each period of measurements. ZnGlu + probiotic modulated CT responses best, especially at day 21.

Figures 2, 3, 4, and 5 show a clear daily rhythmicity of *t*_cloacal_ in all the groups of the broilers on day 28. Rhythm characteristics of *t*_cloacal_ in control group showed a higher mesor, greater amplitude, and delayed acrophases as compared to probiotic and ZnGlu + probiotic groups (Table 2). Specifically, the *t*_cloacal_ value was higher (*P* < 0.05) in control broiler chickens at 17:00–21:00 h and 1:00 h, but the lowest *t*_cloacal_ value was obtained in probiotic‐treated broiler chickens at 11:00 h. ZnGlu‐treated group had lower values of *t*_cloacal_ from 19:00 h to 5:00 h (Fig. 3).

### Variations in cloacal temperature of 35‐day‐old broiler chickens during the hot‐dry season

On day 35, the broilers in all the groups also exhibited clear daily rhythmicity of *t*_cloacal_ with a clear ascent during the photophase and a descent during the scotophase (Figs 2, 3, 4, and 5). The mesor of *t*_cloacal_ in controls was higher (*P* < 0.05) (42.2 ± 0.5°C), with greater (*P* < 0.05) amplitude (1.55 ± 0.02°C) and early acrophase (11:00 ± 1.20 h) as compared to other groups that had lower mesor (41.35–41.7°C), amplitude (0.95–1.30°C), and delayed acrophases (13:00–19:00 h) (Table 2). The extreme minimum‐maximum values obtained in control, ZnGlu, probiotic, and ZnGlu + probiotic groups were as follows: 40.50–43.60°C, 40.40–43.00°C, 40.20–42.50°C, and 40.70–42.60°C, respectively. In general, the probiotic (Fig. 4) and ZnGlu + probiotic (Fig. 5) groups had the lowest *t*_cloacal_ at each hour of recording both during the day and night.

## Discussion

The result showed that the *t*_db_ values obtained during the study period were predominantly outside the thermoneutral zone of 18–24°C (Dei and Bumbie 2011) for mature broiler chickens (days 28–42), reared in a hot tropical climate. The *t*_cloacal_ was measured for 3 days only, 1 week apart, in order to reduce the adverse effects of stress due to handling on the birds, known to increase the body temperature (Edgar et al. 2013). The results of this study showed that thermal environmental conditions of high *t*_db_ (27.00–36.00°C) and relatively high RH (32–70%), prevailing during the hot–dry season, were unfavorable for the rearing of broiler chickens, and that they induced heat stress. Elson (1995) reported that the ideal values of *t*_cloacal_ for broiler chickens vary between 41 and 42°C for a comfortable physiological state, and maximal growth rate and feed intake were observed between the ages of 4 and 8 weeks (Yahav et al. 1995). These values of *t*_cloacal_ concur with the values of 41–41.8°C recorded in this study. Robinson et al. (2016) reported that the ideal t_db_ for broiler chickens within the third week of life is between 26 and 28°C. Similarly, the ideal *t*_db_ for broilers within the fourth and fifth weeks of life is between 12 and 26°C (Sturkie 1976). Wide fluctuations in RH obtained at days 21 (27–77%), 28 (28–70%), and 35 (26–76%) further aggravated the heat stress during the experimental period. The high THI recorded in this study, fluctuating from 25.55 to 34.50°C, was above the recommended THI value of 20.8 for broilers that are < 21 days' old, indicative of severe heat stress (Purswell et al. 2012; Sinkalu et al. 2015b). The high THI may render evaporative cooling mechanism ineffective in the broiler chickens. The results agree with the findings of Purswell et al. (2012), who reported that as THI exceeds approximately 21°C, the performance of birds significantly declines and their body temperature increases. Since heat stress induces excess production of ROS and consequently, oxidative stress (Lara and Rostagno 2013), the high THI obtained in this study strongly suggests that the birds were subjected to heat stress, especially on day 35 and, consequently, oxidative stress. The finding serves as the rationale to mitigate the adverse effects of heat stress by administration of ZnGlu and/or probiotic, which are potent antioxidants (Zhang et al. 2014; Hasan et al. 2015).

The *t*_cloacal_ values obtained in the broiler chickens on day 21, except for probiotic group, did not exhibit daily rhythmicity and had the highest range of 4.40°C, but indicates an inability to maintain a stable body temperature of the broiler chickens at this age. The finding was evidence that the thermoregulatory mechanism of the broiler chickens was more stressed in the control birds than any other group. The lowest *t*_cloacal_ range of 1.4°C recorded in probiotic group showed that probiotic stabilized the *t*_cloacal_ values in the broiler chickens; thus, maintaining the values at this age at a lower range (40.50–41.90°C) than in any other group. It, therefore, appears that the probiotic exerted thermogenic effect on the broiler chickens and modulated the *t*_cloacal_ by synchronizing the circadian rhythm of *t*_cloacal_ at the age of 21 days to normal daily rhythm. Although the acrophase of the *t*_cloacal_ at day 21 did not differ between the groups, the finding that probiotic group showed the lowest *t*_cloacal_ value at the acrophase period and had the smallest amplitude as compared to other groups suggests that probiotic, may be used in synchronizing dysfunctional *t*_cloacal_ rhythms. This finding demonstrates the interrelationship between circadian clocks and metabolism and opens new possibilities for the adoption of nutritional interventions to modulate the circadian clock's function. This requires further investigations. Probiotic facilitates thermogenic activity by increasing the sympathetic activity of the brown and adipose tissues; through enhanced brown adipose tissue thermogenesis in rats (Tanida et al. 2008), and increases expression of thermogenic proteins (uncoupling protein‐2) in mice (Pothuraju et al. 2016). This is a desirable effect. The results of this study showed that probiotic alone, and ZnGlu + probiotic lowered *t*_cloacal_ values, when compared with the controls. Similarly, Egbuniwe et al. (2015) demonstrated a decrease in *t*_cloacal_ values in broiler chickens administered with the antioxidants, betaine and ascorbic acid during the hot‐dry season. Although the mechanism of action of zinc was not elucidated in this study, it may be linked partly to Zn induction of the ultimate antioxidants, metallothioneins; and protection of protein sulfhydryls. It may also be linked to reduction in OH^−^ formation from H_2_O_2_ through the antagonism of redox‐active transition metals, such as iron and copper (Powell 2000), and other ROS generated in excess in heat‐stressed broilers (Gu et al. 2012; Hao et al. 2012). Probiotic, shown to enhance growth in broiler chickens (Aluwong et al. 2013; Zhang et al. 2014), decreased the *t*_cloacal_ values; apparently by improving the intestinal microarchitecture in terms of villus height, and crypt depth in heat‐stressed broiler chickens (Silva et al. 2010). Further investigations are required at the molecular level to elucidate effects of probiotics on broiler chickens, exposed to heat stress during the hot‐dry season.

On day 28, results of *t*_cloacal_ values showed that ZnGlu and probiotic, either singly or in combination significantly reduced *t*_cloacal_ values in broiler chickens, especially starting from 17:00 h to 5:00 h, indicating the beneficial effect of the antioxidants in combating the adverse effect of heat stress on broiler chickens during the hot‐dry season. The result, unlike on day 21, shows a clear ascent in *t*_cloacal_ during the photophase and a decent during the scotophase. Furthermore, the results showed that the lowest *t*_cloacal_ range (1.5–1.7°C) values were obtained in the probiotic and ZnGlu + probiotic groups, indicating that probiotic administration stabilized the *t*_cloacal_ fluctuations and may be most beneficial in normalizing the *t*_cloacal_ fluctuations in broiler chickens at the age of 28 days. By decreasing the *t*_cloacal_ values of the birds, the response of the broiler chickens to administration of antioxidants at this age was beneficial. At this age of 28 days, the thermoregulatory mechanisms of broiler chickens are better developed and their metabolic rate increases rapidly, as evidenced by their rapid growth (Singh et al. 2015). The result of this study showed, for the first time, that the antioxidants, probiotic and ZnGlu have tendency to decreasing the body temperature of the broiler chickens during the last part of photophase period, when the *t*_cloacal_ values are known to rise, starting from 17:00 h (Fig. 4). This observation was supported by the greater amplitude and delayed acrophase (17:00 h) with *t*_cloacal_ well above normal values (42.4 ± 0.5°C) in control broilers, whereas those of probiotics and ZnGlu + probiotics had smaller amplitude, with t_cloacal_ falling within normal reference values (41.7–41.9°C) at an early acrophase period (15:00 h). The present findings showed that the antioxidants were able to offset the adverse effects of thermal load on the broilers at an earlier stage. The antioxidants are, therefore, beneficial and recommended in modulating and combating adverse effects of exposure of broiler chickens to heat stress, particularly starting from day 28.

At the age of 35 days when the broiler chickens were due for slaughter, the *t*_cloacal_ of the control group was well above normal reference values of 40–42°C, whereas probiotic and/or ZnGlu administration decreased *t*_cloacal_ values, starting from 07:00 h to 05:00 h. The finding that probiotic and/or ZnGlu groups had smaller amplitude and delayed acrophases demonstrated that probiotic exerted the most potent effect in reducing the *t*_cloacal_ values of broiler chickens during the hot‐dry season. With increase in age of the broiler chickens, the effect of the antioxidant varied; the lowest reduction in *t*_cloacal_ values was recorded in broiler chickens treated with ZnGlu at 28 days; but at the age of 35 days, the lowest *t*_cloacal_ value was obtained in broiler chickens given only probiotic (Fig. 4). The finding showed that age is a crucial factor in the manifestation of responses of broiler chickens to the stressful thermal environmental conditions of the hot‐dry season. The *t*_cloacal_ responses showed that broiler chickens administered with probiotic had the least *t*_cloacal_ value (41.25 ± 0.05°C), indicating that probiotic exerted the most potent decrease in body temperature in 35‐day‐old broiler chickens.

In general, the overall mean *t*_cloacal_ values recorded in all the groups were within the established normal physiological range (40–42°C) for broiler chickens in the Northern Guinea Savannah zone of Nigeria (Minka and Ayo 2016a). However, at the age of 35 days, especially during the day time, the thermoregulatory mechanism of the birds was adversely challenged, apparently due to increase in metabolic rate and the concomitant effect of heat stress. Thus, the *t*_cloacal_ values obtained at this period were above the normal reference limits. This finding was evidence that the Arbor Acres breed of chickens at day 35, by inability to maintain homeothermy, has not successfully adapted to the unfavorable thermal environmental conditions in the zone during the day time. Thus at 28‐ and 35‐day‐old, it is best to administer ZnGlu and probiotic, respectively to combat the adverse impact of heat stress on broiler chickens during the hot‐dry season. Further studies are required to elucidate the effects of ZnGlu and/or probiotic on other body parameters of broiler chickens; involved in the response of the birds to heat stress, especially the hematological, biochemical, and performance indices. The results of this study, for the first time, demonstrated that ZnGlu and especially probiotic, decreased *t*_cloacal_ responses in broiler chickens, exposed to heat stress during the hot‐dry season; and, in addition, modulated the circadian rhythm of *t*_cloacal_ in broiler chickens. Consequently, the antioxidants may be beneficial in reducing economic losses, often incurred by farmers due to heat stress in broiler production. Furthermore, the age of the birds is crucial in determining the best antioxidant to be administered; with ZnGlu alone being the best before attainment of 28 days' old; and, thereafter, probiotic alone is the best antioxidant to be administered.

In conclusion, probiotic alone decreased *t*_cloacal_ response most, followed by its coadministration with ZnGlu; and the antioxidants may be beneficial in alleviating adverse effects of heat stress on broiler chickens during the hot‐dry season. For this purpose, ZnGlu and probiotic are best administered at the age of 28 and 35 days, respectively.

## Conflict of Interest

The authors declare no conflict of interest.

## References

[phy213314-bib-0001] Ajakaiye, J. J. , J. O. Ayo , and S. A. Ojo . 2010 Effects of heat stress on some blood parameters and egg production of Shika Brown layer chickens transported by road. Biol. Res. 43:183–189. 21031263

[phy213314-bib-0002] Aluwong, T. , H. Fatima , D. Tavershima , K. Mohammed , and A. Joseph . 2013 Effect of different levels of supplemental yeast on body weight, thyroid hormone metabolism and lipid profile of broiler chickens. J. Vet. Med. Sci. 75:291–298. 23100117 10.1292/jvms.12-0368

[phy213314-bib-0003] Ayo, J. O. , S. B. Oladele , A. Fayomi , S. D. Jumbo , and J. O. Hambolu . 1998 Body temperature, respiration and heart rate in the Red Sokoto goat during the harmattan season. Bull Anim. Health Prod. Afr. 46:161–166.

[phy213314-bib-0004] Ayo, J. O. , J. A. Obidi , and P. I. Rekwot . 2011 Effects of heat stress on the well‐being, fertility and hatchability of chickens in the Northern Guinea Savannah zone of Nigeria: a review. ISRN Vet. Sci. 2011:10: https://doi.org/10.5402/2011/838606 10.5402/2011/838606 PMC3658707 23738109

[phy213314-bib-0005] Azad, M. A. , M. Kikusato , T. Maekawa , H. Shirakawa , and M. Toyomizu . 2010a Metabolic characteristics and oxidative damage to skeletal muscle in broiler chickens exposed to chronic heat stress. Comp. Biochem. Physiol. A Mol. Integr. Physiol. 155:401–406. 20036750 10.1016/j.cbpa.2009.12.011

[phy213314-bib-0006] Azad, M. A. K. , M. Kikusato , S. Sudo , T. Amo , and M. Toyomizu . 2010b Time course of ROS production in skeletal muscle mitochondria from chronic heat‐exposed broiler chicken. Comp. Biochem. Physiol. 157:266–271. 10.1016/j.cbpa.2010.07.011 20656050

[phy213314-bib-0007] Chen, X. Y. , P. P. Wei , S. Y. Xu , Z. Y. Geng , and R. S. Jiang . 2013 Rectal temperature as an indicator for heat tolerance in chickens. Anim. Sci. J. 84:737–739. 23634694 10.1111/asj.12064

[phy213314-bib-0008] Chepete, H. J. , E. Chimbombi , and R. P. Tsheko . 2005. Production performance and temperature‐humidity index of Cobb 500 broilers reared in open‐sided naturally ventilated houses in Botswana. Proceedings of the ASAE Annual Meeting; 2005; Beijing, China; p.701

[phy213314-bib-0009] Chowdhury, V. S. , S. Tomonaga , S. Nishimura , S. Tabata , J. F. Cockren , K. Tsutsui , et al. 2012a Hypothalamic gonadotropin‐inhibitory hormone precursor mRNA is increased during depressed food intake in heat‐exposed chicks. Comp. Biochem. Physiol. A Mol. Integr. Physiol. 162:227–233. 22465623 10.1016/j.cbpa.2012.03.009

[phy213314-bib-0010] Chowdhury, V. S. , S. Tomonaga , S. Nishimura , S. Tabata , and M. Furuse . 2012b Physiological and behavioural responses of young chicks to high ambient temperature. J. Poult. Sci. 49:212–218.

[phy213314-bib-0011] Dei, H. K. , and G. Z. Bumbie . 2011 Effect of wet feeding on growth performance of broiler chickens in a hot climate. Br. Poult. Sci. 52:82–85. 21337202 10.1080/00071668.2010.540230

[phy213314-bib-0012] Dzenda, T. , J. O. Ayo , C. A. M. Lakpini , and A. B. Adelaiye . 2011 Seasonal, sex and live weight variations in feed and water consumptions of adult captive African Giant rats (*Cricetomys gambianus*, Waterhouse‐1840) kept individually in cages. Physiol. Rep. 3:1–10. 10.1111/j.1439-0396.2012.01287.x 22404334

[phy213314-bib-0013] Edgar, J. L. , C. J. Nicol , C. A. Pugh , and E. S. Paul . 2013 Surface temperature changes in response to handling in domestic chickens. Physiol. Behav. 119:195–200. 23816981 10.1016/j.physbeh.2013.06.020

[phy213314-bib-0014] Egbuniwe, C. I. , J. O. Ayo , U. M. Kawu , and A. Mohammed . 2015 Cloacal temperature responses of broiler chickens administered with betaine and ascorbic acid during the hot‐dry season. Biol. Rhythm. Res. 46:207–219.

[phy213314-bib-0015] Elson, H. A. 1995 Environmental factors and reproduction Pp. 389–409 in Austic R. E. and Nesheim M. C., eds. Poultry Production. Lea & Febiger, Philadelphia.

[phy213314-bib-0016] Erwan, E. , S. Tomonaga , J. Yoshida , M. Nagasawa , Y. Ogino , D. M. Denbow , et al. 2012 Central administration of L‐and D‐aspartate attenuates stress behaviours by social isolation and CRF in neonatal chicks. Amino Acids 43:969–1976. 10.1007/s00726-012-1272-4 22466305

[phy213314-bib-0017] European Commission . 2007 Council directive 2007/43/EC of 28 June 2007 laying down minimum rules for the protection of chickens kept for meat production. J. Eur. Union 182:19–28.

[phy213314-bib-0018] Franco‐Jimenez, D. J. , and M. M. Beck . 2007 Physiological changes to transient exposure to heat stress observed in laying hens. Poult. Sci. 86:538–544. 17297167 10.1093/ps/86.3.538

[phy213314-bib-0019] Gu, X. H. , Y. Hao , and X. L. Wang . 2012 Overexpression of heat shock protein 70 and its relationship to intestine under acute heat stress in broilers: 2. Intestinal oxidative stress. Poult. Sci. 91:790–799. 22399716 10.3382/ps.2011-01628

[phy213314-bib-0020] Hao, Y. , X. H. Gu , and X. L. Wang . 2012 Overexpression of heat shock protein 70 and its relationship to intestine under acute heat stress in broilers: 1. Intestinal structure and digestive function. Poult. Sci. 91:781–789. 22399715 10.3382/ps.2011-01627

[phy213314-bib-0021] Hasan, S. , M. M. Hossain , I. Alam , and M. E. R. Bhuiyan . 2015 Beneficial effects of probiotic on growth performance and haemato‐biochemical parameters in broilers during heat stress. Int. J. Innov. Appl. Studies 10:244–249.

[phy213314-bib-0022] Lara, L. J. , and M. H. Rostagno . 2013 Impact of heat stress on poultry production. Animals 3:356–369. 26487407 10.3390/ani3020356 PMC4494392

[phy213314-bib-0023] Lin, H. , R. Du , X. H. Gu , F. C. Li , and Z. Y. Zhang . 2000 A study on the plasma biochemical indices of heat stressed broilers. Asian‐Aust. J. Anim. Sci. 13:1210–1218.

[phy213314-bib-0024] Lin, H. , R. D. Malheiros , V. M. B. Moraes , C. Careghi , E. Decuypere , and J. Buyse . 2004a Acclimation of broiler chickens to chronic high environmental temperature. Archiv. Fur. Geflugelkd. 68:39–46.

[phy213314-bib-0025] Meltzer, A. 1983 The thermoneutral zone and resting metabolic rate for broilers. Br. Poult. Sci. 24:471–476. 6667388 10.1080/00071668308416763

[phy213314-bib-0026] Minka, N. S. , and J. O. Ayo . 2013 Effects of antioxidants vitamins E and C on erythrocyte fragility, haemoglobin index and colonic temperature of transported Japanese quails (*Coturnix japonica*). J. Vet. Sci. Technol. 4:1–8.

[phy213314-bib-0027] Minka, N. S. , and J. O. Ayo . 2016a Effect of wet‐cold weather transportation conditions on thermoregulation and the development of accidental hypothermia in pullets under tropical conditions. Int. J. Biometeorol. 60:373–380. 26198381 10.1007/s00484-015-1034-6

[phy213314-bib-0028] Minka, N. S. , and J. O. Ayo . 2016b Effects of cold‐dry (harmattan) and hot‐dry seasons on daily rhythms of rectal and body surface temperatures in sheep and goats in natural tropical environment. J. Circadian Rhythms 14(1):1–11. 30210554 10.5334/jcr.143 PMC5356205

[phy213314-bib-0029] Muniz, E. C. , V. B. Fascima , P. P. Pires , A. S. Carrijo , and E. B. Guimaraes . 2006 Histomorphology of bursa of Fabricius: effects of stock densities on commercial broilers. Braz. J. Poult. Sci. 8:217–220.

[phy213314-bib-0030] Niu, Z. , F. Liu , Q. Yau , and L. Li . 2009 Effects of different levels of selenium on growth performance and immunocompetence of broilers under heat stress. Arch. Anim. Nutr. 63:56–65. 19271551 10.1080/17450390802611610

[phy213314-bib-0031] NRC . 1994 Nutrient Requirements of Poultry. 9th Rev. ed., National Academy Press, Washington, DC.

[phy213314-bib-0032] Piccione, G. , M. Gianesella , M. Morgante , and R. Refinetti . 2013 Daily rhythmicity of core and surface temperatures of sheep kept under thermoneutrality or in the cold. Res. Vet. Sci. 95:261–265. 23523470 10.1016/j.rvsc.2013.03.005

[phy213314-bib-0033] Pothuraju, R. , R. K. Sharma , P. K. Kavadi , J. Chagalamarri , S. Jangra , G. Bhakri , et al. 2016 Anti‐obesity effect of milk fermented by *Lactobacillus plantarum* NCDC 625 alone and in combination with herbs on high fat diet fed C57BL/6J mice. Benef. Microbes 7:375–385. https://doi.org/10.3920/BM2015.0083. 26925603 10.3920/BM2015.0083

[phy213314-bib-0034] Powell, R. S. 2000 The antioxidant properties of zinc. J. Nutr. 130:1447S–1454S. 10801958 10.1093/jn/130.5.1447S

[phy213314-bib-0035] Purswell, J. L. , C. D. Dozier , A. A. Olarenwaju , J. D. Davis , H. Yin , and R. S. Gates . 2012. Effect of temperature‐humidity index on live performance in broiler chickens grown from 49 to 63 days of age. ASABE Conference Presentation, 9th International Livestock Environment Symposium; Valencia, Spain; Jul 8‐12, 2012; 9 p

[phy213314-bib-0036] Refinetti, R. , G. Cornélissen , and F. Halberg . 2007 Procedures for numerical analysis of circadian rhythms. Biol. Rhythm. Res. 38:275–325. 23710111 10.1080/09291010600903692 PMC3663600

[phy213314-bib-0037] Robinson, O. H. , F. F. T. Iida , A. O. S. Jairo , B. M. Luciano , S. O. R. Keller , and M. G. G. Lina . 2016 Thermal environment in two broiler barns during the first three weeks of age. R. Bras. Eng. Agric. Ambiental. 20:256–262.

[phy213314-bib-0038] Scheele, C. W. , derVan Hel W. , M. W. A. Verstegen , and A. M. Henken . 2014 Climatic environment and energy metabolism in broilers Pp. 217–226 in Verstegena M. W., Henken A. M. Eds. Energy metabolism in farm animals: effects of housing, stress and disease. Martinus Nijhoff, Dordrecht.

[phy213314-bib-0039] Silva, V. K. , J. D. Torre da Silva , R. A. Gravena , R. H. Marques , F. H. Hada , and V. M. Barbosa de Moraes . 2010 Yeast extract and prebiotic in pre‐initial phase diet for broiler chickens raised under different temperatures. R. Bras. Zootec. 39:165–174.

[phy213314-bib-0040] Singh, K. A. , K. T. Ghosh , C. D. Creswell , and S. Haldar . 2015 Effects of supplementation of betaine hydrochloride on physiological performances of broilers exposed to thermal stress. Open Access Anim. Physiol. 7:111–120.

[phy213314-bib-0041] Sinkalu, V. O. , and J. O. Ayo . 2008 Diurnal fluctuations in rectal temperature of Black Harco pullets administered with vitamins A and C during the hot‐dry season. Int. J. Poult. Sci. 7:1065–1070.

[phy213314-bib-0042] Sinkalu, V. O. , J. O. Ayo , A. A. Abimbola , and J. E. Ibrahim . 2015a Effects of melatonin on cloacal temperature and erythrocyte osmotic fragility in layer hens during the hot‐dry season. J. Appl. Anim. Res. 43:52–60.

[phy213314-bib-0043] Sinkalu, V. O. , J. O. Ayo , A. B. Adelaiye , and J. O. Hambolu . 2015b Ameliorative effects of melatonin administration and photoperiods on diurnal fluctuations in cloacal temperature of Marshal broiler chickens during hot‐dry season. Int. J. Biometeorol. 59:79–87. 24748234 10.1007/s00484-014-0826-4

[phy213314-bib-0044] Soleimani, A. F. , A. Kasim , A. R. Aliman , A. Meimandipour , and I. Zulkifli . 2010 Ileal endogenous amino acid flow of broiler chickens under high ambient temperature. J. Anim. Physiol. Anim. Nutr. (Berl) 94:641–647. 20050954 10.1111/j.1439-0396.2009.00951.x

[phy213314-bib-0045] Sturkie, P. D. 1976 Regulation of body temperature Pp. 343–379 in Whittow G. C., ed. Avian Physiology. Springer‐Verlag, New York.

[phy213314-bib-0046] Tanida, M. , J. Shen , K. Maeda , Y. Horii , T. Yamano , Y. Fukushima , et al. 2008 High‐fat diet‐induced obesity is attenuated by probiotic strain *Lactobacillus paracasei* ST11 (NCC2461) in rats. Obesity Res. Clin. Pract. 2:159–169. 10.1016/j.orcp.2008.04.003 24351773

[phy213314-bib-0047] Tao, X. , and H. Xin . 2003 Acute synergistic effects of air temperature, humidity, and velocity on homeostasis of market‐size broiler. Trans. Am. Soc. Agric. Eng. 46:491–497.

[phy213314-bib-0048] Vale, M. M. , D. S. Moura , I. A. Naas , and D. F. Pereira . 2010 Characterisation of heat waves affecting mortality rates of broilers between 29 days and market age. Braz. J. Poult. Sci. 12:279–285.

[phy213314-bib-0049] Yahav, S. , S. Goldfeld , I. Plavnik , and S. Hurwitz . 1995 Physiological responses of chickens and turkeys to relative humidity during exposure to high ambient temperature. J. Therm. Biol 20:245–253.

[phy213314-bib-0050] Zhang, X. , Y. Zhao , Q. Chu , Z. Y. Wang , H. Li , and Z. H. Chi . 2014 Zinc modulates high glucose‐induced apoptosis by suppressing oxidative stress in renal tubular epithelial cells. Biol. Trace Elem. Res. 158:259–267. 24591003 10.1007/s12011-014-9922-x

